# Plant MIR2911 in honeysuckle is effective against SARS-CoV-2 variant

**DOI:** 10.1016/j.virusres.2025.199583

**Published:** 2025-05-13

**Authors:** Chen-Huang Shen, Huey-Pin Tsai, Chih-Chieh Chou, Chun-Sheng Yeh, Tsen-Hsuan Yen, Wen-Long Huang, Meilin Wang, Michael W.Y. Chan

**Affiliations:** a Department of Urology, Ditmanson Medical Foundation Chiayi Christian Hospital, Chiayi, Taiwan; b Department of Biomedical Sciences, National Chung Cheng University, Min-Hsiung, Chia-Yi, Taiwan; c Department of Medical Laboratory Science and Biotechnology, College of Medicine, National Cheng Kung University, Tainan 701, Taiwan; d Department of Pathology, National Cheng Kung University Hospital, College of Medicine, National Cheng Kung University, Tainan 701, Taiwan; e Epigenomics and Human Diseases Research Center, National Chung Cheng University, Min-Hsiung, Chia-Yi, Taiwan; f Center for Innovative Research on Aging Society (CIRAS), National Chung Cheng University, Min-Hsiung, Chia-Yi, Taiwan; g Department of Chinese Medicine, Dalin Tzuchi Hospital, The Buddhist Tzuchi Medical Foundation, Chiayi, Taiwan; h Department of Microbiology and Immunology, School of Medicine, Chung-Shan Medical University, Taichung, Taiwan; i Department of Clinical Laboratory, Chung-Shan Medical University Hospital, Taichung, Taiwan; j Research Center for Precision Environmental Medicine, Kaohsiung Medical University, Kaohsiung, Taiwan

**Keywords:** COVID-19, SARS-CoV-2, MIR2911, JCPyV CLP

## Abstract

The COVID-19 pandemic remains a global health challenge, with the emergence of new SARS-CoV-2 variants complicating treatment and prevention efforts. MIR2911, a plant-derived microRNA from honeysuckle, has shown antiviral activity against the original strain of SARS-CoV-2. Here, we investigate its efficacy against the Delta variant (B.1.617.2). Computational analysis using RNAhybrid identified multiple MIR2911 recognition elements (MREs) in the Delta variant genome. Reporter assays confirmed that MIR2911 binds these MREs, significantly reducing luciferase activity. In Vero E6 cells infected with the Delta variant, MIR2911 suppressed RdRP RNA expression and viral replication in a dose-dependent manner, as evidenced by plaque assays. To enhance its therapeutic potential, we developed a novel delivery system using JCPyV CLP, a virus-like particle capable of transducing genetic material. CLP-mediated delivery of MIR2911 not only further improved its antiviral efficacy, reducing RdRP RNA expression and viral plaque formation with minimal toxicity, but also significantly enhanced MIR2911 transfection efficiency compared to conventional liposome-based methods. These findings highlight the potential of MIR2911, delivered via JCPyV CLP, as a promising therapeutic strategy for combating SARS-CoV-2 replication, including the highly virulent Delta variant.

## Highlights

• MIR2911 targets SARS-CoV-2 Delta variant.
• Reporter assays confirm MIR2911 binding to viral RNA.
• MIR2911 inhibits viral replication in infected cells.
• JCPyV CLP enhances MIR2911 delivery and efficacy.
• CLP-mediated MIR2911 shows low toxicity and strong antiviral potential.

## 1 Introduction

The COVID-19 pandemic continues to be a global public health crisis, with over 750 million confirmed cases and >7 million deaths as of July 2024 (WHO 2024). Despite the availability of vaccines (Khoury et al., 2021) and antiviral therapies (Andrews et al., 2024), the emergence of new variants and the potential side effects associated with current treatments necessitate the exploration of alternative therapeutic options.

Honeysuckle (*Lonicera japonica*), a climbing vine in the Caprifoliaceae family, has long been used in traditional Chinese medicine for its antiviral, anti-inflammatory, and immune-modulating properties. It is rich in bioactive compounds such as chlorogenic acid, luteoloside, and flavonoids. One of its most notable applications in modern medicine is its potential role in treating SARS-CoV-2, the virus responsible for COVID-19. Recent studies have highlighted the potential of MIR2911, an atypical miRNA derived from the cleavage of the 16S ribosomal RNA (rRNA) of honeysuckle (Shang et al., 2011), as a promising antiviral agent against SARS-CoV-2, both in vitro and in human studies (Zhou et al., 2020). This effect may be attributed to MIR2911’s unique GC-enriched nucleotide composition, which allows it to directly target and suppress viral gene expression, thereby inhibiting viral replication and accelerating viral clearance in infected patients (Zhou et al., 2020). This discovery has generated significant interest in the use of *Lonicera japonica* as a natural therapeutic agent against SARS-CoV-2. Additionally, its anti-inflammatory effects may help mitigate the cytokine storm associated with severe COVID-19 cases, providing further therapeutic benefits.

While these findings are promising, they focused on the original strain of the virus. Given the ongoing evolution of SARS-CoV-2, we aimed to investigate whether MIR2911 could exert similar antiviral effects against other variants, particularly the Delta strain.

## 2 Material and methods

### 2.1 Luciferase reporter assays

Human embryonic kidney HEK293 cells (2 × 10^5^ per well) were seeded into 6-well plates, one day before transfection. 5 μM of MIR2911(Omicsbio Co., Ltd, Taiwan) or control (scramble) were cotransfected with 1 μg of Mir-glo (Promega, Madison, WI) luciferase reporter plasmids with 3′-UTR-containing miRNA recognition elements (MRE) of ORF1ab region. Cells were then lysed after 24-h transfection, and luciferase activities measured by the Dual-Luciferase Reporter Assay System, with Renilla as the transfection control (Promega), according to the manufacturer’s instructions.

### 2.2 JCPyV CLPs preparation

The *E.coli*-expressing plasmid△pFlag-JCPyV1 bearing the VP1 gene of JCPyV was transformed into *E.coli* (JM109) with ampicillin selection. JCPyV CLPs were generated by recombinant VP1 protein in *E.coli* expression system. CLPs were purified by 20 % sucrose cushion centrifugation, CsCl velocity gradient centrifugation and 10–30 % sucrose gradient centrifugation. Particle-containing fractions with hemagglutination activity were collected and dialyzed against Tris-buffered saline. CLPs were concentrated with a Centricon filter (Millipore, Billerica, MA)10 μg CLPs were mixed with 2 μg miR2911 and was incubated for 10 min at 37 °C. Osmotic shock was achieved by diluting the mixture with 200 μl of distilled water and incubated for 30 min at 37 °C. Then mixed with 10x TBS and incubated for 20 min at room temperature.

### 2.3 Transfection and plaque assay

Vero-E6 cells (1.6 × 10^6^) were seeded overnight and transfected with control, 5 μM, or 10 μM of MIR2911 using a transfection reagent (Lipofectamine 3000; 20 μL/flask). At 24 h post-transfection, cells were infected with Delta strain (full-length genome sequence GISAID No:EPI_ISL_17,952,197) of SARS-CoV-2 virus at a multiplicity of infection (MOI) of 1.2 (1.96 × 10^6^ Copies RNA/1.6 × 10^6^ Cell) in a Biosafety-3 Laboratory. The reverse-transcription quantitative polymerase chain (RT-qPCR) reaction and plaque assay were performed as previously described (Case et al., 2020) to determine the viral load in the culture supernatant.

### 2.4 Comparison of transfection efficiency

MIR2911-Cy5 (5 μM) was delivered into Vero-E6 cells (1.6 × 10^6^) using either transfection reagent (Lipofectamine 3000) or JCPyV CLPs as described above. After 2 h, cells were visualized using Upright Fluorescence Microscope (Axio Imager A2, ZEISS, Oberkochen, Germany), the fluorescence intensity was also quantified by Multi-Moude Microplate Reader (excitation 630 nM, emission 670 nM, ApectraMax iD3, Molecular Device, San Jose, CA)

### 2.5 Determination of viral load

Viral RNA was extracted from the supernatant was using Viral RNA Mini Kits (Qiagen GmbH, Hilden, Germany). The qRT-PCR was performed as previously described (Lee et al., 2022). The viral titer in the culture supernatant was determined by plaque assay using Vero-E6 cells (initially seeding 2 × 10^5^ /well) in 24-well culture plate as previously described (Case et al., 2020). After 7 days of SARS-CoV-2 infection, the supernatant was removed and fixed with 10 % formalin for 30 min, inactivated with UV light for 30 min, then removed the formalin and stained with 0.1 % crystal violet for 30 min.

### 2.6 Bioinformatic and statistical analysis

Identification of MIR2911 recognition elements (MREs) was performed by RNAhybrid (Kruger and Rehmsmeier, 2006). Statistical significance was determined using GraphPad Prism Version 5.0 software for Windows (GraphPad Software, La Jolla, CA, USA). The Student’s *t*-test was used to compare parameters of different groups.

## 3 Results and discussion

**Fig. 1:**
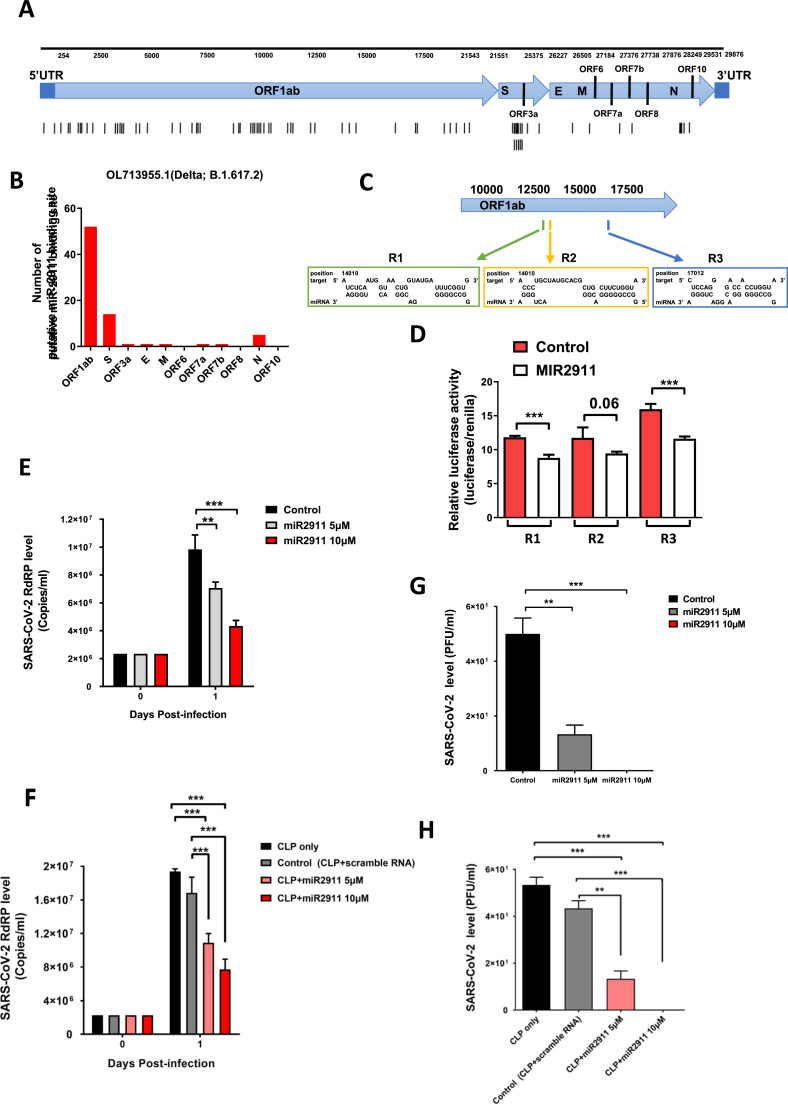
Computation prediction of miR2911 binding site on (A) Delta strain (B.1.617.2) of SARS-CoV-2 genome using RNAhybrid. (B) Quantitative analysis of the number of putative MIR2911 binding sites at each transcripts of the virus are shown. (C) The putative MIR2911 MREs at ORF1ab regions and the corresponding sequences are shown. (D) Those sequences were cloned in the 3′UTR of the pGL3 luciferase plasmid and transfected together with pRL plasmid into 293 cells. The relative luciferase activity with or without MIR2911 are shown. Effect of MIR2911 on the expression of RdRP RNA level and vital SARS-CoV-2 in Vero E6 cells. Vero E6 cells were first transfected with 5 or 10 μM of MIR2911 using lipofectamine 3000 (E, G) or CLP-containing MIR2911 (F, H), followed by infection with Delta strain (EPI_ISL_17,952,197) of SARS-CoV-2 in Vero E6 cells. Expression of RdRP RNA level (E, F) was determined by qRT-PCR; while vital viral quantification was determined by plaque assay (G, H).

In this study, we first analyzed the genomic sequence of the Delta variant (B.1.617.2) using RNAhybrid (Kruger and Rehmsmeier, 2006) and confirmed the presence of several MREs (Fig 1A,B). To verify MIR2911’s ability to bind these MREs, we constructed luciferase plasmids containing the 3′UTR regions of these viral sequences. Transfection of MIR2911 significantly suppressed luciferase activity, indicating that MIR2911 could bind effectively to the viral MREs (Fig 1C, D). We next tested the antiviral effects of MIR2911 by transfecting it into Vero E6 cells, followed by infection with the Delta variant. Post-infection analysis showed a strong expression of RdRP RNA level, a key viral gene, which was significantly reduced by MIR2911 in a dose-dependent manner (Fig 1E). Plaque assays further confirmed that MIR2911 effectively inhibited viral replication (Fig 1G).

We also explored a novel delivery system for MIR2911 using JCPyV CLP, a virus-like particle that we have previously shown to efficiently transduce genetic material without the need for native viral genes (Chou et al., 2024). JCPyV CLP can carry nucleic acids up to 9.4 kb, providing an efficient delivery method with minimal toxicity compared to traditional liposome-based transfection (Lin et al., 2019). Our results showed that CLP-mediated delivery of MIR2911 not only suppressed RdRP RNA level expression (Fig 1F) but also significantly reduced viral plaque (Fig 1H) formation in a dose-dependent manner, further validating the potential of this delivery system for therapeutic purposes.

**Fig. 2:**
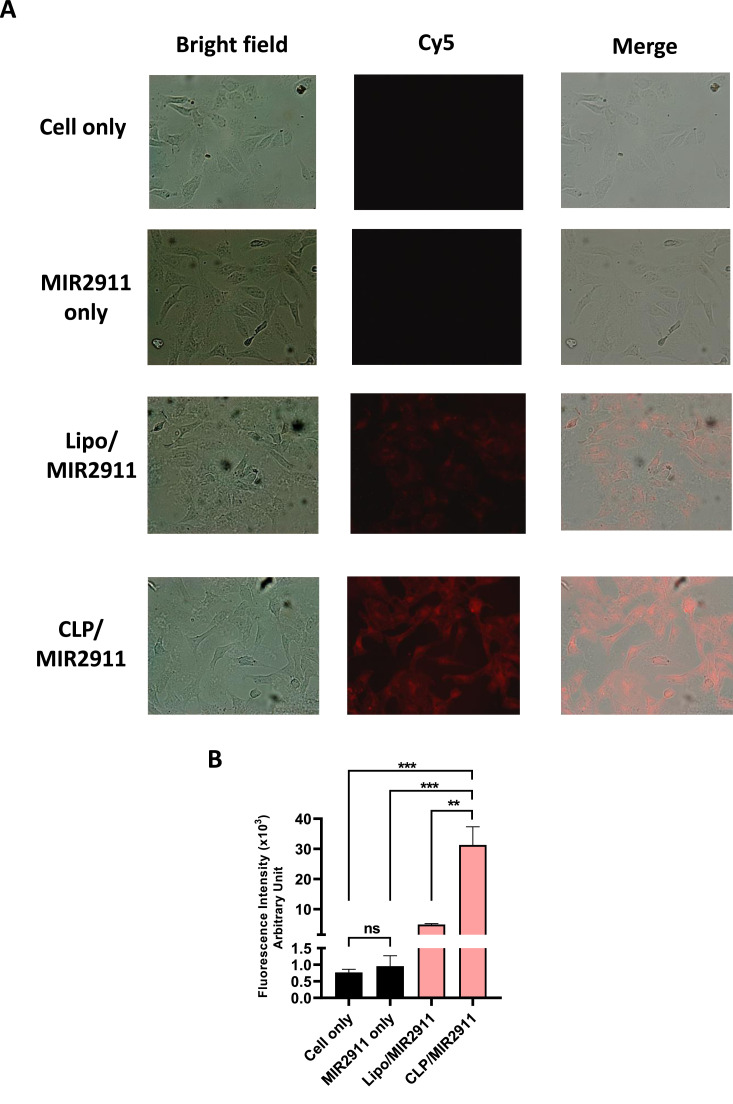
Comparison of transfection efficiency between liposome and JCPyV CLP. Cy5-labelled MIR2911 was delivered into Vero-E6 cells using either a transfection reagent (Lipofectamine 3000) or JCPyV CLPs. Cells without MIR2911 (cell only) and cells treated with MIR2911 without a transfection reagent (MIR2911 only) served as controls. (A) Cells were visualized under a fluorescence microscope (400x magnification). Left panel: phase-contrast bright field; middle panel: Cy5 fluorescence; righr panel: merged image. (B) Histogram showing fluorescence intensity of MIR2911 as quantified by spectrophotometry. ***P < 0.001, **P < 0.01, ns: not significant.

To confirm that the enhanced inhibition of viral replication is due to more efficient delivery of MIR2911 by JCPyV CLP, we compared the transfection efficiency of Cy5-labeled MIR2911 using either lipofectamine or CLP. As expected, the fluorescence intensity of MIR2911 was significantly higher in cells transfected with CLP compared to those transfected with lipofectamine (Fig. 2A, B).

## 4 Conclusion

In conclusion, our findings demonstrate that MIR2911, a plant-derived microRNA from honeysuckle, effectively inhibits SARS-CoV-2 replication, including the Delta variant. This is consistent with previous reports supporting the therapeutic potential of honeysuckle in treating COVID-19 (Du et al., 2021; Yeh et al., 2021). The mechanism underlying this antiviral effect is likely the binding of MIR2911 to viral MREs, leading to the suppression of key viral genes like RdRP. Our study also highlights the potential of JCPyV CLP as a delivery vector for MIR2911, offering a novel and efficient method for enhancing its therapeutic effects. Further research is needed to explore the broader applicability of MIR2911 in combating other emerging SARS-CoV-2 variants.

## Ethics approval

This study is approved by the IRB of Ditmanson Medical Foundation Chiayi Christian Hospital, Taiwan (CYCHIRB2023036).

## Consent for publication

All authors read the final manuscript and agreed to publish it.

## Funding

This study was supported by grants from the National Science and Technology Council, Taiwan (NSTC 111–2923-B-194–001-MY3, 112–2740-B-006–003-, 113–2314-B-194–002-MY3), Ditmanson Medical Foundation Chiayi Christian Hospital, Taiwan (R107–001 and R109–39–3), 10.13039/501100004844National Cheng Kung University Hospital (NCKUH-11108001) and the Research Center for Precision Environmental Medicine, 10.13039/501100004694Kaohsiung Medical University, Kaohsiung, Taiwan from The Featured Areas Research Center Program within the framework of the Higher Education Sprout Project by the Ministry of Education (MOE) in Taiwan and by Kaohsiung Medical University Research Center Grant (KMU-TC113A01) to MWYC.

## Author statement

This short communication has not been previously published and is not under consideration for publication elsewhere. We have no conflicts of interest to disclose, and all authors have approved the final version of the manuscript.

## CRediT authorship contribution statement

**Chen-Huang Shen:** Writing – review & editing, Funding acquisition, Conceptualization. **Huey-Pin Tsai:** Resources, Methodology, Funding acquisition. **Chih-Chieh Chou:** Writing – review & editing, Investigation. **Chun-Sheng Yeh:** Investigation. **Tsen-Hsuan Yen:** Investigation. **Wen-Long Huang:** Investigation. **Meilin Wang:** Writing – review & editing, Writing – original draft, Methodology, Investigation, Conceptualization. **Michael W.Y. Chan:** Writing – review & editing, Writing – original draft, Investigation, Funding acquisition, Conceptualization.

## Declaration of competing interest

The authors declare no conflict of interest.

## Data Availability

All data have been shown
